# Identification of potent inhibitors of microtubule affinity regulating kinase for inhibition of tau hyperphosphorylation

**DOI:** 10.1186/1750-1326-8-S1-P28

**Published:** 2013-09-13

**Authors:** Alison Levy, Michelle Newman, Janet Brownlees, Denise Harding, Joanne Osborne, Stephen Lewis, Kevin Gillen, Gareth Hall, Chido Mpamhanga, Andy Merritt, Debra Taylor, Ed Mclver

**Affiliations:** 1 Centre for Therapeutics Discovery, MRC Technology, London, UK; 2 Department of Biochemistry, University of Leicester, Leicester, UK

## 

The four MARK (microtubule affinity regulating kinase) kinases (MARKs 1-4) are an evolutionary conserved group of proteins, which belong to the AMPK-related protein kinase family and are involved in the regulation of cell polarity. MARKs are highly expressed in brain and were first identified through their ability to phosphorylate and regulate the dissociation of microtubule-associated proteins (MAPs) from microtubules[1]. In particular, MARK phosphorylation of the MAP tau at KXGS motifs within the microtubule-binding domain was shown to dissociate tau from microtubules leading to de-stabilisation of the microtubule network. The MARK-mediated phosphorylation of tau has also been linked to the formation of neurofibrillary tangles (NFTs) observed in Alzheimer’s Disease (AD) and other neurodegenerative diseases collectively referred to as tauopathies[2,3]. Based on available evidence it is proposed that inhibitors of MARK could prevent the formation of hyperphosphorylated tau protein and thus be useful for the treatment of Alzheimer’s and other neurodegenerative diseases. We identified a potent chemical series of MARK inhibitors and conducted a medicinal chemistry lead optimisation program to explore SAR and improve the affinity, kinase selectivity and ADME profiles of our lead compounds. This work was aided by a combination of first *in silico* modelling followed by X-ray crystallography. We also developed and used a high content imaging assay to monitor tau phosphorylation at the Ser^262^ MARK epitope in primary rat neurons, to demonstrate the effect of MARK inhibitors in a physiologically relevant cellular environment. The aim was to identify a potent and selective ATP-competitive MARK inhibitor that could be used for ‘proof of concept’ studies in transgenic mice expressing human tau and potentially as a starting point for a drug development program.
